# In Vitro Culture, Genetic Transformation and the Production of Biopharmaceuticals in Microalgae

**DOI:** 10.3390/ijms26083890

**Published:** 2025-04-20

**Authors:** Aneta Gerszberg, Ludmiła Kolek, Katarzyna Hnatuszko-Konka

**Affiliations:** 1 Department of Molecular Biotechnology and Genetics, Faculty of Biology and Environmental Protection, University of Lodz, Banacha 12/16, 90-237 Lodz, Poland; 2 Institute of Ichthyobiology and Aquaculture in Gołysz, Polish Academy of Science, Zaborze, Kalinowa St 2, 43-520 Chybie, Poland

**Keywords:** microalgae, in vitro culture, genetic engineering, pharmaceuticals

## Abstract

Microalgae represent a promising platform for the synthesis of recombinant proteins, particularly in the context of biopharmaceutical applications. Their unique combination of eukaryotic cellular machinery and prokaryotic-like simplicity offers several advantages, including the ability to perform complex post-translational modifications, rapid growth rates, and cost-effective culture conditions. Advances in genome sequencing, genetic engineering tools, and omics technologies have significantly enhanced the feasibility and efficiency of using microalgae for therapeutic protein production. These advancements, coupled with the development of well-established transformation methods and optimized vectors, have enabled the successful expression of various biopharmaceuticals, ranging from vaccines to enzymes. Here, the main stages and current status of the production of exogenic recombinant proteins dedicated to human therapy are presented.

## 1. Introduction

According to various classifications, microalgae may be defined as single-cell eukaryotic microorganisms (nearly 800,000 species), as cited by Olasehinde and colleagues [1], or as uni- or simple multicellular organisms encompassing both prokaryotic (cyanobacteria—blue-green algae) and eukaryotic species (green algae and diatoms), as presented by Stavridou et al. [2]. Regardless of the division they represent, microalgae are capable of thriving in wild, demanding environments, rapidly reacting to changes in external conditions. Today, they attract significant attention primarily as biomass for biologically active food and feed additives or as a widely understood substrate for the isolation of valuable bioactive substances. The latter includes scenarios where microalgae serve as either a rich natural source of nutrients or a platform for the synthesis of heterologous compounds. Their ability to rapidly increase biomass (most species can double their biomass in a maximum of 24 h) and their flexible adaptation of cellular metabolism provide strong arguments for the use of microalgae in pharmaceutical biotechnology. This is especially relevant given that the technologies used for biomass production are generally environmentally safe and require relatively small amounts of water [3]. Additionally, one of the undeniable advantages of microalgae is their biodiversity, which allows them to serve as heterogeneous sources of diverse compounds [1].

Microalgae produce nutrients and biologically active substances, including proteins, polysaccharides, lipids, carotenoids, vitamins, and secondary metabolites. They are secreted during different growth phases of algal cells. Many of these compounds have been proven to exhibit antitumor, antioxidant, antihypertensive, antibacterial, antiviral, neuroprotective, and immunostimulating properties. Some also demonstrate prebiotic properties influencing gastrointestinal microbiota. Given these properties, it is no surprise that they attract significant attention from researchers in medicine, pharmacology, and cosmetology. This interest is further justified by the fact that several species are certified for human consumption, particularly those from the *Arthrospira* and *Chlorella* genera, which are recognized as functional foods in accordance with the Food and Drug Administration (FDA) [4]. The FDA’s “Generally Recognized As Safe” (GRAS) status requires time-consuming and expensive safety tests; therefore, only a few microalgal species have been recognized as edible: *Dunaliella bardawil*, *Porphyridium cruentum*, *Crypthecodinium cohnii*, *Arthrospira platensis*, *Haematococcus pluvialis*, *Euglena gracilis*, *Chlamydomonas reinhardtii*, *Chlorella vulgaris*, *Chlorella protothecoides*, and *Auxenochlorella protothecoides*. The GRAS microalgae species are classified as safe also because they do not share any mutual pathogens with humans [5,6,7]. As overconsumption continues to negatively impact the environment and with the global population estimated to reach 9.8 billion by 2050, the increasing need for nutritious proteins and health-promoting ingredients is expected to grow significantly [8]. Consequently, the role of edible microalgae in providing valuable bioproducts for both food and medicine is anticipated to expand [4]. This offers a dual benefit, as edible microalgae not only provide essential nutrients but also serve as an efficient and cost-effective platform for synthesizing heterologous proteins, with GRAS species specifically facilitating the production of edible vaccines. Therefore, the development of efficient procedures for microalgae cultivation and genetic transformation is crucial to harness their full potential for novel applications.

The interest in microalgae as the platform for the production of heterologous recombinant proteins is relatively new in comparison to their use as a source of food or feed. Microalgae represent a promising potential for competing with plant, yeast, mammalian, insect and bacterial hosts. They were reported to be capable of producing a wide spectrum of therapeutic compounds, such as antibodies, subunit vaccines, antibiotics, clotting factors, hormones, neurotoxic and hepatotoxic compounds, enzymes, and others with therapeutic applications, which are produced by chemical methods with certain constraints [9].

This review presents an updated outlook on the main stages and current status of the production of exogenic recombinant proteins dedicated to human therapy, with a particular focus on genetic tools and transformation procedures. The isolation of microalgal strains, cultivation requirements and applied cultivation protocols are characterized only as necessary points of the construction of transgenic microalgal biofactories. We do not focus on metabolic engineering, native gene expression alterations, or gene editing techniques (except for presenting examples of algal species with the greatest potential as biopharmaceutical biofactories). In our article, we adopted the perspective that microalgae constitute a heterologous group, encompassing both microscopic unicellular and simple multicellular organisms, including prokaryotic (cyanobacteria—blue-green algae) and eukaryotic (green algae and diatoms) representatives.

## 2. Isolation and Purification of Microalgal Strains

Scientists have developed methodologies that enable the isolation of microalgal strains from diverse ecosystems, including ice-covered regions, geothermal springs, freshwater and brackish water bodies, rivers, marine environments, reservoirs, wastewater sources, rocks, saline lakes, coastal zones, and soil. A crucial step after sample collection—where microalgae are mixed with various bacteria and fungi—is the removal of unwanted microorganisms to obtain axenic algal cultures for further research and applications. This multi-stage process typically involves a series of dilutions, inoculations, and the implementation of antibacterial and antifungal treatments. However, to ensure the acquisition of pure and uncontaminated microalgal species, certain pre-isolation procedures must be followed: (1) upon transfer to the laboratory, nutrient supplementation is applied to natural samples to enhance microalgal proliferation; (2) before the isolation process, contaminants are removed from microalgal cells using techniques such as vortexing, sonication, and surfactant treatment [10].

Two commonly used inoculation methods for microalgae isolation are the pour plate method and the streak/spread plate method. The pour plate method is employed for algae that do not grow on the agar surface. In this technique, microalgae dilution is achieved by suspending cells in poured agar, where they become immobilized and serve as the foundation for new colonies. Conversely, the streak/spread plate method enables the isolation of individual microalgal colonies by inoculating the agar surface with a spreader or loop, similar to bacterial inoculation techniques [10].

Another simple and effective method for obtaining pure algal strains is serial dilution. In this approach, axenic culture growth occurs in the tube with the highest dilution. To further ensure the purity of cultures, additional procedures are often applied in parallel, such as UV radiation, micromanipulation, or density centrifugation.

A further technique, known as single-cell isolation by micropipette, utilizes the capillary method. In this procedure, individual cells (serving as inoculum) are extracted from the sample using a micropipette and transferred to a sterile drop of water. This process is repeated until a single cell is isolated, with the entire operation monitored under a microscope [10].

However, conventional methods (e.g., the streak/spread plate method and serial dilution) are often labor-intensive and not sufficiently efficient for obtaining purified strains. Therefore, more effective and convenient alternatives are being explored. Recently, Ali and Mirza [11] proposed a simpler, more cost-effective, and faster method for isolating microalgal strains. This approach involves the use of alginic acid beads, in which individual dispersed algal species are trapped and subsequently cultured in separate wells of 96-well plates.

Similarly, Chen et al. [12] developed a simple, disposable paper-based device for the separation of individual microalgal cells. The device was fabricated within minutes using a standard laser printer without requiring lithographic tools or a cleanroom. Its functionality is based on capillary action, allowing for easy operation with a laboratory micropipette without the need for a microscope. Furthermore, the device can be adapted for automated sampling combined with high-throughput analysis.

Another effective technique for isolating specific microalgal strains is Fluorescence-Activated Cell Sorting (FACS). This method identifies specific cell populations based on their optical properties, including light scattering and fluorescence emission [13]. While FACS enables the rapid isolation of uncontaminated, single-species microalgal cultures, its main drawbacks include high cost and operational complexity.

One of the most recent advances in single-cell microalgae isolation is the use of zone electrophoresis in megabore capillary tubes. This method enables cell separation based on size, shape, and morphology within a uniform electric field at a low separation voltage. Compared to serial dilution, it offers a relatively faster separation process. Additionally, the throughput can be increased by simultaneously utilizing multiple megabore capillary tubes [14]. Notably, unlike capillary electrophoresis or dielectrophoresis—which rely on high voltages that may cause cell damage or deformation—the described method preserves cell viability and integrity.

Following isolation, various culturing media are used to cultivate microalgae. Commonly employed media include Blue-Green Medium (BG11), f/2, TNBR, Bold Basal Medium (BBM), and Tris-Acetate-Phosphate Medium (TAP) [11,14,15,16,17,18,19,20].

## 3. How Are Microalgae Cultivated?

Recently, interest in algae cultivation has increased significantly among scientists due to their unique properties, particularly their ability to synthesize valuable bioactive compounds used in various industries and their rapid biomass growth [21,22]. However, one of the greatest limitations to large-scale microalgae production is the creation and development of cost-effective production systems. Consequently, recent studies have focused on optimizing the conditions for microalgae cultivation to enhance biomass growth rates and increase the production of desired compounds under artificial conditions [20,23].

Microalgae have a crucial advantage: they can be cultivated on a large scale under strictly controlled conditions. This is made possible by photobioreactors—devices that provide optimal conditions for the growth of organisms whose main energy source is solar radiation. These include not only algae but also mosses, cyanobacteria, and purple bacteria. Through photosynthesis, these organisms convert sunlight, water, and carbon dioxide into oxygen and organic matter, such as sugars. In the artificial environment of a photobioreactor, this process is meticulously controlled [24,25].

### 3.1. Parameters Affecting Biomass Growth

The level of biomass accumulation or the productivity of biologically active substances is determined by the strain of microalgae. These parameters are influenced by many factors, including the composition of the medium, pH, temperature, lighting, and, finally, the growth phase and harvesting method [19,26,27,28]. The medium for growing microalgae should primarily contain inorganic ingredients such as nitrogen, phosphorus, and carbon, whose content varies depending on the strain being cultivated. Moreover, for proper microalgae growth, the presence of trace elements in the medium is essential. These elements include Mg, S, Ca, Na, Cl, Fe, Zn, Cu, Mo, Mn, B, and Co, with particular importance attributed to Mg, S, and Fe [22].

### 3.2. Hydrogen Potential

Hydrogen potential (pH) is crucial for algae cultivation, as it determines the solubility and, therefore, the availability of individual components in the medium. Additionally, pH directly affects the algae themselves. The hydrogen potential depends on several factors, such as the amount of dissolved carbon dioxide, the composition and buffer capacity of the medium, temperature, and the metabolic activity of the cells [24]. The optimal pH range for algae growth varies depending on the type of algae. In most cases, ideal growth is observed within a pH range of 6–8 or even up to 11 [19]. There are also documented cases of intensive growth of *Galdieria sulphuraria* and *Chlamydomonas acidophila* biomass at extremely low pH values ≥3 [29]. An effective way to control pH changes while simultaneously increasing microalgae biomass in culture is to enrich the culture by injecting CO_2_ or aerating it by pumping it into atmospheric air [30,31,32].

### 3.3. Temperature

The second critical factor influencing the production of microalgae biomass and internal compounds is temperature, primarily due to its impact on enzymatic reactions [33,34]. The optimal growth temperature for the most commonly used mesophilic microalgae ranges from 20 °C to 35 °C [33,35]. However, this range can vary depending on the substrate used and the strain being cultured [28]. Interestingly, some strains exhibit remarkable resistance to stress caused by high temperatures. Varshney et al. [17] isolated two novel green algal strains, *Asterarcys quadricellulare* and *Chlorella sorokiniana*, which demonstrated tolerance to temperatures exceeding 43 °C, alongside resistance to elevated levels of CO_2_ and NO_2_. In contrast, culture temperatures below 16 °C are generally unsuitable for photosynthesis and growth, leading to inhibited growth rates or reduced biomass production. Similarly, temperatures above 35 °C are often lethal for many microalgae species [35].

Temperature conditions not only influence growth but also correlate with the production of biocompounds, such as lipids, in microalgae. For instance, increasing the culture temperature to 30 °C in an engineered strain of *Phaeodactylum tricornutum* enhanced both biomass production and fatty acid content [34]. Conversely, in the case of *Xanthonema hormidioides*, a lower temperature (20 °C) promoted the accumulation of unsaturated fatty acids, whereas a temperature of 30 °C proved lethal for this species [36]. Several studies have also demonstrated the significant impact of temperature on the accumulation of carotenoids in various microalgae species [27,28,37].

### 3.4. Light

Another important factor influencing the growth of microalgae is light. Optimal conditions for cell growth depend on three key parameters: light intensity, wavelength, and photoperiod. Photosynthesis, the fundamental process driving microalgae growth, is defined as the production of organic compounds from inorganic matter within chlorophyll-containing cells under the influence of light [38]. Among these parameters, light intensity plays a particularly crucial role, as it is directly linked to the efficiency of the photosynthesis process. As the availability of light—whether natural or artificial—increases, the rate of photosynthesis in microalgae correspondingly rises until it reaches a saturation point. Beyond this point, excessive light exposure leads to photoinhibition, a phenomenon where the efficiency of photosynthesis declines due to damage to the photosynthetic apparatus [39].

Many studies indicate that a key factor influencing the production of microalgae biomass and biocompounds is the use of different light spectra and radiation intensities [27,28,38]. Photosynthetically active radiation (PAR) for microalgae ranges from 400 to 700 nm [38]. A study by Katam et al. [40] demonstrated that blue light at an intensity of 300 μmol/m^2^/s and red light at an intensity of 100 μmol/m^2^/s were the most effective in promoting algal cell growth and enhancing photosynthetic performance. These findings align with those of Diaz-MacAdoo et al. [27], who observed that highly intense red and blue light significantly boosted microalgae biomass and lutein production. In addition to light intensity, the duration of light/dark cycles (L/D) plays a crucial role in enhancing microalgae biomass and biological compound production [39]. In the cultivation of various algae species, including marine and freshwater types, it is common to employ continuous lighting or alternating light and dark cycles, including pulsed lighting [21,23,39]. The use of photoperiods during cultivation is well justified, as it corresponds to the nature of photosynthesis—a process consisting of light-dependent photochemical reactions (occurring during the light phase) and light-independent reactions (occurring during the dark phase).

Optimal lighting conditions promote increased microalgae biomass growth, resulting in higher cell density within the culture. While this is desirable, it is important to note that increased cell density can limit light penetration, thereby reducing photosynthesis efficiency and overall culture productivity [24]. Mixing cultures has proven to be an effective strategy for addressing this challenge. Mixing not only maintains high cell concentrations but also ensures uniform access to light and substrate components while enhancing gas exchange. Moreover, mixing improves photosynthesis by facilitating carbon dioxide capture from the gas phase and oxygen release from the liquid phase [26]. Research by Sanchez et al. [15] showed that mixing significantly influenced the growth of *Isochrysis galbana*, leading to a two-fold increase in biomass compared to static culture conditions. However, excessive turbulence caused by vigorous agitation can negatively impact microalgae, as their cell walls are sensitive to hydromechanical forces. Overmixing can result in cell damage and reduced photosynthetic rates. Therefore, when planning the cultivation of a specific species, it is essential to select an appropriate cultivation system that balances agitation intensity with the biological requirements of the algae.

### 3.5. Algal Cultivation Techniques

The wide range of algae cultivation techniques offers varying levels of control over growth and product yield, which are closely tied to capital and operational costs [24]. Algae cultivation can be categorized into three basic methods: heterotrophic, photoautotrophic, and mixotrophic. The photoautotrophic method is the most common, leveraging algae’s ability to harness solar energy and inorganic carbon to produce chemical energy. In contrast, the heterotrophic method allows microalgae to grow in both light and dark conditions by utilizing organic carbon. However, this method is limited to a few biotechnologically significant species, such as *Chlorella* spp., *Chlorella protothecoides*, *Crypthecodinium cohnii*, *Galdieria sulphuraria*, *Neochloris oleoabundans*, and *Nitzschia laevis* [41]. The third method, mixotrophy, combines the advantages of photoautotrophy and heterotrophy, enabling microalgae to switch their nutritional modes depending on the availability of energy sources [21,24].

Considering the photosynthetic capacity of algae and their cultivation methods, two primary systems have been developed for large-scale production: open and closed systems. Open systems typically consist of external shallow ponds arranged in loops, with rotors providing turbulence [41]. Their primary advantage is low cost and ease of scalability. However, these systems are prone to contamination by microorganisms and other microalgae. Additionally, process control is challenging due to reliance on changing weather conditions, further complicating cultivation efforts [25].

To address the limitations of open systems, closed devices known as photobioreactors (PBRs) have been developed. These systems ensure higher reliability, productivity, and control over cultivation conditions. Based on their structure, PBRs are classified as flat-panel or tubular designs, with the latter configured in serpentine or branched systems and positioned vertically or horizontally [23,25]. Typically constructed from transparent materials such as glass or plastic, PBRs involve higher production costs. Closed systems allow for continuous cultivation, enabling high-quality biomass production and efficient photosynthesis. However, increased culture density can hinder light penetration due to the shadow effect, where light does not reach deeper layers of the culture [24]. To overcome this obstacle, newer solutions were tested in the form of an annular-column photobioreactor with internal LED illumination to achieve biomass density [20]. Additionally, an internally illuminated mirror photobioreactor was used to improve microalgae production through homogeneous light distribution [22]. While closed systems reduce fluid evaporation, removing excess oxygen—a byproduct of photosynthesis—remains a challenge. Moreover, the complexity of PBR design complicates scaling up. Consequently, cultivation in closed systems is more expensive than in open systems [25]. For this reason, closed systems are predominantly used for producing high-value biological compounds, such as recombinant proteins, pigments, and polyunsaturated fatty acids [20,41,42].

## 4. Genetic Engineering of Microalgae for Heterologous Therapeutic Recombinant Proteins Production

As mentioned, microalgae hold significant scientific and industrial interest due to their potential for genetic manipulation applications. Genetic engineering of microalgae can lead to accelerated growth rates, optimized biofuel production, enhanced productivity, and the acquisition of valuable compounds. Among these, the production of recombinant proteins with therapeutic properties appears to attract the greatest attention. This is fully justified by three important facts: proteins constitute 50% to 70% of the fresh weight of microalgae; recombinant proteins cover more than 60% of commercialized biopharmaceuticals; and the global market for protein-based therapeutics is projected to reach $566.66 billion by 2030 [6,43,44]. According to the definition provided by Bolaños-Martínez and colleagues [45], biopharmaceuticals are “molecules specifically produced under biotechnological processes based on genetically engineered organisms used as an expression host”. In our paper, these molecules are more specifically defined. We focused on exogenous recombinant peptides and proteins intended for human therapy (excluding natural products of microalgae).

These highly specific and effective therapeutic proteins, produced in biological systems, must exhibit high purity, ensure full clinical functionality, allow for simple and cost-effective large-scale industrial manufacturing, and be free from hazardous contaminants. The biological characteristics of the target protein must be thoroughly understood, as they dictate the selection of recombinant hosts, production methods, equipment, facilities, downstream applications, and overall manufacturing strategies, which must be carefully designed and optimized to ensure the production of high-quality biopharmaceuticals with guaranteed safety and efficacy in human therapy. This should be carefully considered at the very early stage, prior to the construction of the expression cassette [46]. In terms of host organisms, microalgae must compete with well-described and method-equipped model organisms or economically important species, such as plants, bacteria, mammals, fungi, and insects. Currently, most therapeutics for human therapy are recombinant glycoproteins synthesized in mammalian cells (e.g., human embryonic kidney 293; Chinese hamster ovary, CHO cells; baby hamster kidney, BHK21, cells; murine myeloma cells, NS0 and Sp2/0) [6,47]. They offer a cellular environment closely resembling the native (human) one, complex post-translational modifications (notably glycosylation) and dedicated folding machinery. However, mammalian cells are difficult to manipulate genetically, expensive to grow and maintain, and in the case of more complex proteins, they cannot be replaced by low-cost systems like bacteria or yeast. Among bacteria, *Escherichia coli* is the first-choice host for the synthesis of prokaryotic proteins, as well as certain eukaryotic proteins. However, *E. coli* lacks the capacity to perform most post-translational modifications and to correctly fold complex proteins (e.g., eukaryotic membrane proteins, large multi-domain assemblies, multi-subunit complexes, or proteins containing multiple disulfide bonds). Yeast species (e.g., *Pichia pastoris*, *Saccharomyces cerevisiae*, *Kluyveromyces lactis*) can, in turn, carry out some modifications essential for proper protein processing. However, yeast introduce non-homogeneous hypermannosylated structures into glycosylated proteins, which differ from mammalian ones and may induce antigenicity. Similarly, post-translational modifications limit the use of other eukaryotic systems like insects or plants. Insect cells, although one of the most widely used systems for heterologous biopharmaceuticals, cannot introduce complex-type N-glycans [48]. It is also an obstacle in the case of plant-based systems. There are differences in the nature of N-glycan composition (the absence of α1–6 fucose, sialic acid and glucose compared to mammalian cells). The introduction of alternative substituents into protein structures may, for example, affect the half-life or immunogenicity of biopharmaceuticals [49]. Moreover, the situation concerning GM plants is still not always favourable.

Microalgae, with their rapid growth and cost-effective culture conditions, combine eukaryotic cellular machinery that is capable of performing post-translational modifications with prokaryotic-like simplicity. Along with their high biodiversity and high protein content, this has led to growing interest among pharmaceutical biotechnologists. As a result, microalgae have been successfully engineered to produce a variety of recombinant proteins, including vaccines, cytokines, antibodies, growth factors, and enzymes (examples of biopharmaceuticals dedicated to human therapy expressed in microalgal systems are presented in Table 1).

Undoubtedly, the metabolic characteristics, ecofriendly properties, and economic benefits of microalgae make them a viable alternative for the synthesis of recombinant proteins. Furthermore, notable advancements in genome editing technologies (e.g., ZNFs, TALENs and CRISPR-Cas9, which enhance recombinant expression and knock out sequences that impede this process), genome sequencing, and all “the (micro)algae omics” technologies (genomics, proteomics, transcriptomics) have further increased algal productivity [66,67]. The list of sequenced microalgal genomes and sequencing projects currently in progress is presented in a paper by Kumar and colleagues [68]. To the best of the authors’ knowledge, more than 190 microalgal genomes have been almost fully sequenced, with the majority completed over the last 20 years. Among these, green microalgae, led by the most researched *Chlamydomonas reinhardtii* (genome sequence published in 2007), have the highest representation [2,69]. It is worth noting that, to date, six versions of the *C. reinhardtii* reference genome have been released using various technologies (Sanger, Sanger+, PacBio + Illumina, and Nanopore + Illumina). *Chlamydomonas* has become the primary model species in both plant and cell biology and now serves as a reference for the rapidly expanding field of algal biotechnology [70]. This clearly illustrates that harnessing omics-based methods, combined with statistical analysis, has brought a more comprehensive understanding of algal genetic makeup and molecular mechanisms, thereby supporting genetic engineering and the synthesis of biopharmaceuticals. Referring to genome sequencing, it should be noted, however, that despite recent advancements, the full potential of microalgae remains largely unrealized due to the limited understanding of their regulatory networks and metabolic pathways. This is partly because knowledge derived from synthesized genomes and other “omics” technologies cannot always be extrapolated to lesser-studied microalgae. The main challenge lies in the insufficient sequence similarity of a large fraction of genes to well-characterized species. Consequently, assigning a metabolic function to the products of potentially homologous genes remains difficult [71].

*Chlamydomonas reinhardtii*, *Haematococcus pluvialis*, *Dunaliella salina* (representing *Chlorophyceae*), *Phaeodactylum tricornutum* (*Bacillariophyceae*, diatom), *Nannochloropsis oculata* (*Eustigmatophyceae*), and *Chlorella* sp. (e.g., *C. vulgaris*, *C. sorokiniana*) (*Trebouxiophyceae*) are primary sequenced species used for protein therapeutics synthesis [6,72]. This was made possible by well-established transformation techniques for both nuclear and chloroplast genomes (the genetic toolkits developed for *Chlamydomonas reinhardtii* and *Phaeodactylum tricornutum* are the most advanced). Namely, microparticle bombardment, electroporation, polyethylene glycol-mediated transformation, and *Agrobacterium*-mediated transformation, all of which have been demonstrated to be able to introduce recombinant DNA into microalgae genomes [72]. However, although there is a wide variety of techniques and nuclear and chloroplast *cis*-regulatory elements available, the majority of biopharmaceuticals have been expressed in the former system [73,74]. Both the nucleus and chloroplasts possess advantages and disadvantages concerning biopharmaceutical expression. Therapeutic proteins frequently undergo post-transcriptional and post-translational modifications, which are essential for their biological function. These modifications include alternative splicing, site-specific proteolysis, proper protein folding, glycosylation, and the formation of disulfide bonds. Such requirements favour nuclear genome engineering, as it enables protein secretion, targeted expression of foreign proteins within specific cellular compartments, and the aforementioned post-translational modifications. However, the algal nucleus exhibits lower expression efficiency (due to integration mode—non-homologous end joining). Chloroplast transformation, on the other hand, has garnered significant attention due to its inherent advantages. These include enhanced and stable expression levels of the gene of interest (GOI), achieved through precise integration into specific loci within the chloroplast genome (two of the most frequent integration sites are the psbA and psbH), thereby eliminating position effects and gene silencing [73,74,75]. Additionally, chloroplast engineering allows for multi-gene expression under the control of a single promoter, a feature attributed to the prokaryotic origin of chloroplasts. However, this bacterial ancestry also results in the absence of post-translational modifications. Furthermore, in chloroplast-based expression, biopharmaceuticals remain confined within the organelle [73,74,76]. Regardless of the transformed genome, the level of protein synthesis remains a challenge. Among other factors, this may be linked to differences in codon usage. Variations in codon usage have been detected not only between microalgal species but also within the same species, between the chloroplast and nuclear genomes, and among organelles. For example, adenine and thymine more frequently appear in the third position of chloroplast codons, whereas guanine and cytosine are more prevalent in nuclear codons. Therefore, codon optimization of heterologous genes can improve the expression of biologics [73,75].

### 4.1. Tools for Genetic Engineering of Microalgae

Genetic engineering is of central importance in molecular farming. Scientific experience has identified key factors and steps that need to be analysed for the overall development of heterologous biopharmaceuticals. Ramos-Vega and coworkers [63] discuss them in the context of vaccines. However, most of these aspects are of significant importance for the construction of transgenic organisms producing biologics in general. These factors/steps include the following: 1) microalgae species/strain, displaying desired biological features; 2) genetic construction, encompassing, among others, target protein, transformed genome, chosen type of transformation (stable or transient) cassette elements and expression vector, codon optimization, post-translational modifications, and secretion or subcellular localization and retention; 3) transformation method; 4) methods of transgene and product detection; 5) protein recovery and purification, followed by testing of biological activity. An appropriate example of the development of heterologous biologics for human therapy, according to the scheme presented above, is the construction of an *Arthrospira* (Spirulina)-based vaccine (PfCSP edible vaccine against malaria). The subsequent steps, including microalgal species selection, vector design, Spirulina transformation, recombinant protein purification, and preclinical and clinical trials, have been outlined across the continuum of studies by Jester et al. [77] and Saveria et al. [61], based on experiments conducted within the framework of the Lumen Bioscience Inc. Startup.

In most cases, the modification of the algal genome involves well-known engineering tools. It is important to clarify the categories of tools that can be distinguished. Apart from transformation techniques, which will be discussed in a separate section, a tool can also address (1) a particular microalgae species with known sequences and methodological support, (2) a genetic vector understood as a set of regulatory elements, or (3) a regulatory element itself. Regarding the tool, species *C. reinhardtii* has been briefly introduced. Its dominance stems, among other factors, from the relatively short period between obtaining initial transformants and evaluating protein expression, as well as well-established modification techniques for both genomes [73,74].

Another primary host for foreign biopharmaceutical synthesis is the marine diatom species *Phaeodactylum tricornutum*. It is one of the most examined diatoms, with a complete genome sequenced. Found in brackish to saline waters worldwide, *P. tricornutum* is polymorphic, displaying at least three main morphotypes: oval, triradiate, and fusiform [78]. In Europe, at least eight companies currently cultivate the species (e.g., Simris, Hammenhög, Sweden, and AlgaTechnologies, Ketura, Israel), producing up to four tons of dry biomass per year. *P. tricornutum* is used for the production of fucoxanthin and eicosapentaenoic acid (EPA). Additionally, both *P. tricornutum* extract and EPA-rich oil extract have been submitted for approval as novel foods by the European Union, with evaluations still underway [79]. The species has gained prominence in biotechnology for a range of applications, including as a host for recombinant protein expression, thanks to its biosynthetic abilities, resilient adaptation to a wide range of culture media, and rapid growth rates [80]. *P. tricornutum* attracted further interest in 2012 when Hempel and Maier [81] introduced a modification to the expression system by eliminating the endoplasmic reticulum retention signal. This change allowed the antibody against the Hepatitis B Virus surface protein to be secreted into the culture medium by the diatom. Since the species typically does not secrete many of its proteins, recombinant proteins can be easily purified, resulting in highly cost-effective production [81]. In 2017, Hempel and colleagues [50] successfully used the engineered *P. tricornutum* to produce monoclonal IgG antibodies against the nucleoprotein of the Marburg virus MARV (a highly pathogenic virus that, similar to the Ebola virus, causes severe hemorrhagic fever). The correctly assembled antibodies were secreted into the medium and demonstrated functional activity [50]. It would undoubtedly be tempting to use such a system as a dual-purpose product, providing both antibodies and food. However, this is not permitted at this stage, as, to the best of our knowledge, *P. tricornutum* does not have functional food/GRAS status. Undoubtedly, the example of engineered *P. tricornutum* raises the question of whether dual benefits are possible. Could genetically modified microorganisms serve as both a source of secreted biologics and biomass? In the aforementioned experiment, Hempel et al. utilized the endogenous inducible nitrate reductase (NR) gene promoter and terminator, which is considered an excellent tool for inducible transgenic manipulation [50]. Consequently, the NR promoter creates the potential conditions under which engineered cells producing antibodies could simultaneously be recovered for biomass production. After collecting the medium containing antibodies and establishing a new microalgae culture, protein synthesis could be terminated by transferring the utilized biomass to a fresh, non-inducing medium. An additional passage might be required to eliminate any residual secreted antibodies present in the medium. However, it would be essential to demonstrate that no functional antibodies remain in the biomass, even after synthesis termination. Nevertheless, the concept is intriguing and offers the possibility of dual benefits.

To remain economically viable, recombinant protein production must achieve high expression rates. In this context, other “types of tools” are considered, such as vectors and expression cassettes. Typical expression constructs consist of two key cassettes: one for selecting transformants and the other for driving transgene expression. Distinct selection systems operate for chloroplast and nuclear genomes. These include selection markers based on photoautotrophic growth, metabolic enzymes, antibiotic resistance, and herbicide resistance for chloroplast expression, as well as auxotrophic growth, antibiotic resistance, and herbicide resistance for nuclear expression [73].

A gene of interest (in this case, a heterologous therapeutic protein sequence) is equipped with appropriately selected promoters, enhancers, terminators, and/or reporter genes. A novel approach harnessing the aforementioned *cis*-regulators along with other factors and steps (e.g., strains, media, introns, vectors) included in the general scheme of recombinant protein production is presented in an excellent paper by Rajput et al. [82]. For optimal protein production, the most crucial *cis*-elements are the promoters and the 5′/3′ regulatory mRNA untranslated regions (UTRs), with UTRs playing a key role in mRNA stability [44,83]. Due to the potential for significant evolutionary divergence between a heterologous protein and the target microalgae, successful transgene expression often requires the use of native promoters and corresponding UTRs from the host species (if available). However, a review of recombinant protein expression in microalgal systems indicates that native, heterologous, hybrid, and synthetic promoters have all been utilized. Examples of both native and heterologous promoters used for protein expression in plastidic and nuclear microalgal systems are provided in Table 2.

Typically, promoters and 5’UTRs from photosynthetic genes (*atpA*, *psaA*, *rbcL*, *psbD*, and *psbA*) or *16*S RNA (both endo- and exogenic), are used to regulate transgene expression in chloroplasts [83]. The inclusion of a 3′UTR from various genes is also necessary; however, it has minimal impact on transcript accumulation and foreign protein expression. For example, human growth hormone (hGH) was shown to have biological activity in vitro after being expressed in *C. reinhardtii* chloroplasts under the control of the psaA and atpA promoters and the rbcL 3′-UTR [84]. For nuclear genomes, the use of strong endogenous promoters is also advised. Among the native regulatory elements, the RBCS2 promoter, which drives the expression of the Rubisco small subunit, and the PSAD promoter, which regulates an abundant chloroplast protein in the Photosystem I complex, were shown to effectively drive nuclear transgene expression in *C. reinhardtii* [73]. The nucleus of this model species was also successfully transformed with an expression cassette for human lactoferrin (hLF) under the control of the exogenous CaMV35S promoter [52].

Transgene expression was further enhanced when hybrid promoters were developed. The RBCS2 promoter was combined with the HSP70A promoter (HSP70A-RBCS2 fusion—AR) and the glutamate dehydrogenase gene (*GDH2*) promoter with the acyl carrier protein gene (*ACP2*) promoter (GDH2-ACP2 fusion—GA), with GA showing a sevenfold higher expression than AR. However, whether in the microalgal nucleus or chloroplast, the accumulation of foreign proteins remains limited. The highest expression levels observed in chloroplast-transformed genes reached up to 5% of the total soluble protein in *C. reinhardtii* [67]. To address the shortage of strong promoters, researchers have turned to synthetic biology approaches, whose achievements are comprehensively reviewed by Gupta et al. [67].

**Table 2:** Examples of endo- and exogenous promoters used in microalgal systems.

Host Species	Promoter	Target Genome	References
	Endogenous promoters		
*N. gaditana*	constitutive promoters of HSP90 and EPPSII	nuclear	[85]
*N. gaditana*	nitrate-inducible NR promoter	nuclear	[86]
*N. oceanica*	constitutive bidirectional promoters of the NR/NT gene, of Ribi and of CP1/2	nuclear	[87,88]
*N. oceanica*	constitutive rbcL promoter	plastidic	[89]
*N. oceanica*	elongation factor promoter	nuclear	[90]
*N. oceanica*	tubulin promoter	nuclear	[90]
*N. oceanica*	nitrate reductase promoter	nuclear	[90]
*T. pseudonanas*	silicon-repressible SIT promoter	nuclear	[91]
*C. vulgaris*	CvNDI promoter	nuclear	[56]
*P. purpureum*	tubulin promoter	nuclear	[92]
*P. tricornutum*	constitutive rbcL promoter	plastidic	[93]
*P. tricornutum*	constitutive HASP1 promoter	nuclear	[94]
*P. tricornutum*	nitrate-inducible NR promoter	nuclear	[95]
*C. reinhardtii*	CrGPDH3 promoter	nuclear	[96]
*C. reinhardtii*	light-inducible psbA promoter	plastidic	[83]
*C. reinhardtii*	atpA promoter	plastidic	[73]
*C. reinhardtii*	psbD promoter	plastidic	[73]
	Exogenous promoters		
*C. vulgaris*	CaMV35S promoter	nuclear	[97]
*C. vulgaris*	CaMV35S promoter	nuclear	[98]
*C. ellipsoidea*	ubiquitin 1 promoter	nuclear	[99]
*C. sorokiniana*	CaMV35S promoter	nuclear	[100]
*P. tricornutum*	ClP1 promoter of a diatom-infecting virus	nuclear	[101,102]
*C. reinhardtii*	LIP promoter	nuclear	[103]
*C. reinhardtii*	antifreeze protein (AFP) promoter	nuclear	[104]

Vectors designed for microalgae modification often combine elements characteristic of both eukaryotic and prokaryotic organisms. This allows for the construction of expression cassettes in bacterial species, such as *Escherichia coli*. The prokaryotic backbone of the vector (e.g., pBluescript, CAMBIA plasmids) typically contains the origin of replication, antibiotic markers, and multiple cloning sites. For example, binary expression vectors pCAMBIA-1304 and pCAMBIA-1301, mostly employed for plant transformation, were used for *D. pseudosalina* and *C. reinhardtii,* respectively [52,105]. Vector construction can be simplified by using the Gateway cloning system [2]. This system utilizes compatible recombination “att-sites” to facilitate in vitro transfer of DNA sequences between plasmids (entry and destination vectors), bypassing ligase-mediated cloning. The use of the Gateway^®^ recombination system in the construction of transformation vectors for microalgae was first reported in 2014 in *Chlamydomonas reinhardtii* (chloroplast genetic engineering) [106]. Since then, a number of Gateway destination vectors have been constructed for the analysis of promoter specificities, protein interactions, protein localization, and other algal genetic studies. Gateway technology became a foundation for the development of the MoClo (Modular Cloning) toolkit with a standard syntax. It includes 119 functionally validated nuclear gene fragments from *C. reinhardtii* [107]. Clearly, vector sequences have been engineered to facilitate the process of microalgal transformation. Their construction reflects the strategy for recombinant protein production, e.g., genome to be transformed (chloroplast, nucleus), planned compartments of protein subcellular localization (chloroplast, ER, cytoplasm), detection and purification methodologies.

### 4.2. Methods of Genetic Transformation

So far, a number of different methods of introducing transgenes into microalgae have been developed, including *Agrobacterium*-mediated, nanoparticles or silicone-carbide fibers, particle bombardment, glass bead agitation, agitation with the presence of surfactant (e.g., PEG), electroporation [42,51,108,109,110]. Nevertheless, due to its simplicity and high efficiency, electroporation is the most widely used [111]. In the case of *Chlamydomonas reinhardtii*, this technique allows for a much larger number of transformants and low false-positive results compared to the other methods mentioned above [112]. Generally, this method involves treating cells with an electrical impulse to generate micropores in the cell wall, which allows for the uptake of exogenous DNA (transgene). In order to maximize the transformation efficiency, individual parameters of the method are optimized. An example is the research of Muňoz et al. (2018) in which the optimal parameters for electroporation, such as voltage setting, concentration of microalgae cells, lighting intensity and the size of the inserted DNA fragment, were determined for four species of algae (*Chlamydomonas reinhardtii*, *Chlorella vulgaris*, *Neochloris oleoabundans* and *Acutodesmus obliquus*) [113]. On the other hand, Poweda-Huertes et al. [114] examined the influence of cell cycle synchronization and the addition of adjuvant (saponin) on the transformation efficiency in two species of microalgae (*Nannochloropsis oceanica* and *Phaeodactylum tricornutum*). The research conducted by Naser et al. [115] showed that not only the voltage but also the number of pulses is important to increase the transformation efficiency. The results obtained by the authors may be a guide for designing conditions for the transformation of other economically important microalgae strains. Electroporation has been successfully used to obtain vaccine subunit production in *N. oceanica* cells [111].

Even if electroporation remains the first-choice method for transforming microalgae, in certain situations, researchers use other methods, including PEG-mediated, particle bombardment or *Agrobacterium* methods. The success of the biolistic method is influenced by many factors, including the density of the microalgae cells, the target organelle, the amount of DNA on the particles, the number of particles coated with DNA, the kinetic energy of the particles imparted by the appropriate helium pressure, and the cells’ capacity for regeneration after particle-induced damage. Recently, highly effective particle bombardment was demonstrated in the case of *Fistulifera solaris* under optimized conditions, with results 37 times better than conventional particle bombardment [115]. However, a less complicated glass bead method using only vortexing microalgae cells in tubes with glass beads was successfully used to transform *C. reinhardtti*, obtaining expression of the human IL-29 gene in its cells [42]. Although this method is simple and cheap because it does not require the use of advanced equipment, it will only work in the case of algae strains that have cell wall defects or if they are treated with enzymes at the initial stage.

*Agrobacterium*-mediated transformation, a simple and reliable delivery method routinely used in the case of genetic modifications of higher plants, has also been implemented for the transformation of microalgae. As in the case of *Agrobacterium*-mediated plant transformation, many factors play a role in the efficiency of microalgae transformation, including the type of co-cultivation medium, acetosyringone concentration, co-culture duration, the microalgal strain [100]. In the case of *C. reinhardtti*, this method obtains a transformation frequency comparable to that obtained using the biolistic method [110]. One of the main advantages of this approach is the stable integration of the introduced fragment into the recipient genome. However, the main drawback is the fact that the non-selective allele is frequently truncated. Recently, this method has been successfully used to produce a fully functional recombinant protein (human interferon (IFN)) with antiviral and anticancer activity in *C. reinhardtti* cells [51].

Although the method of PEG-mediated transformation is easy and simple to perform and is characterized by high efficiency or lack of species specificity, it is seldom used for genetic modification of microalgae. A significant obstacle to the widespread use of the PEG method for microalgae transformation is the presence of a cell wall. Some studies indicate the successful adaptation of this procedure to the transformation of cell-wall-deficient microalgae (e.g., *Cyanidioschyzon merolae*) as well as those where the cell wall has been removed via enzymes (e.g., cellulase, macerase, pectinase, and hemicellulose) [116]. Parameters affecting the effectiveness of PEG transformation include the presence/absence of a cell wall, cell size, surfactant concentration, duration of agitation and the form of DNA used (linear or circular). In order to improve the transformation efficiency for selected microalgal strains, various parameters for the PEG transformation approach are continuously optimized. Recently, Guo et al. [109] tested some crucial parameters (e.g., antibiotic concentration, growth stage, amount of transformed vector, linearization of the vector, and duration of low-intensity illumination) for improving the transformation efficiency of *Haematococcus pluvialis* protoplasts.

Since the transformation rate is a limiting factor in chloroplast biotechnology, research is being conducted on the use of new nanotechnology-based delivery approaches as tools for modifying algal chloroplasts. Newkirk et al. [117] investigated the impact of polyethylenimine-coated single-walled carbon nanotubes (PEI-SWCNT) as delivery vehicles for DNA to *C. reinhardtti* chloroplasts. The study showed that both the size of the PEI-SWCNT charge and the size of the polymer affect the uptake of particles by chloroplasts. On the other hand, Kim et al. [118] investigated an approach using DNA carriers, gold nanoparticles (AuNPs). Single-stranded DNA (ssDNA) was covalently attached to the surface of the AuNPs, and spherical nucleic acid (SNA) was formed. The uptake of SNA by microalgae (*Ochromonas danica*) cells took place without the involvement of external stimuli. The presence of SNA was found in both the vacuole and cytoplasm without affecting photosynthetic activity or growth of the microalgae. This promising finding may suggest that SNA can be used as a tool for gene modification. Given the many challenges of SNA technology, including versatility, efficiency of particle uptake, and long-term estimated toxicities, it still requires much additional research.

Transformation methods commonly used for eukaryotic microalgae have been successfully adapted to the transformation of prokaryotic microalgae [105]. However, since a few cyanobacteria species, including *A. platensis*, demonstrate the ability to take up DNA from the surrounding environment without the presence of surfactant, the natural transformation method is used to genetically modify them [77]. In this case, transformation efficiency depends on several factors, including the physical and chemical characteristics of the cyanobacterial species/strain, the growth phase of the culture, and the use of DNase inhibitors. In addition, the concentration of the introduced DNA and its length and form (single- or double-stranded) are also important. Although the detailed mechanism of the aforementioned process is not entirely clear, analyses carried out on several species of cyanobacteria revealed that type IV pili facilitate DNA uptake [119].

## 5. Proteins Extraction Methods Form Microalgae

The process of protein production, including biopharmaceuticals, on an industrial scale involves the cultivation stage of microalgae, followed by their extraction and purification processes to obtain the desired product. The selection of the appropriate species/strain of microalgae for therapeutic protein production is crucial from the very beginning. During cultivation, microalgal cells are exposed to various types of stress (e.g., agitation, aeration), which can negatively affect the growth rate of their biomass and viability [15]. Therefore, the most suitable species are those whose cell walls are as durable as possible, such as green algae (e.g., *Chlorella*, *Haematococcus pluvialis*) [120]. The nature of the cell wall is also important for downstream processing steps, including protein recovery. In terms of composition and the molecular bonds that shape its structure, the cell wall shows significant variability among microalgal species. In this regard, the cell wall of microalgae (e.g., *C. vulgaris*, *H. pluvialis*, *N. oculata*) is significantly more robust compared to cyanobacteria (e.g., *A. platensis*) [121]. Moreover, the composition and thickness of the cell wall of microalgae are also influenced by the environmental conditions and cultivation parameters applied [122].

Due to the fact that algae differ significantly in terms of the structure of the cell wall, it is not possible to develop a single universal protein isolation protocol. The selection of the appropriate procedure is, therefore, influenced by several important factors, including the strain/species of microalgae, the thickness of the cell wall and even the shape of the cell. In addition, when choosing a procedure, such parameters as the type of isolated protein should also be considered. From the point of view of the cost-effectiveness of obtaining therapeutic proteins produced in algal cells, optimizing the extraction procedure should be associated with a thorough examination of such key factors as energy consumption, the presence and stability of metabolites, toxicity, and scalability of the method. Over the years, a wide range of different protein extraction methods has been developed based on the assessed criteria mentioned above [123,124,125]. The main obstacle to accessing the components of the microalgal cell is the rigidity of the cell wall. To overcome this difficulty, many techniques for breaking the continuity of the cell wall have been developed. To date, conventional methods for recovering proteins from microalgae rely on chemical (acid, base, and enzymatic treatment), physical (drying, sonication, pulse electric field), and mechanical (cell homogenization, bead milling) processes to disrupt the cell wall’s continuity. The efficacy of these methods varies with respect to the degree of cell wall disintegration, as well as the release of target components and the required energy input. Undoubtedly, the choice of the method used also has a significant impact on the quality and efficiency of the final product [124,125].

Typically, proteins are extracted using different solvents (e.g., bases, acids) as well as surfactants, which significantly increase the permeability of the cell wall. The most commonly used for microalgae cell wall disruption and protein solubilization is the use of alkaline treatment [121]. On the other hand, with regard to extraction by enzymatic techniques, these employ a wide range of cell wall-degrading enzymes (e.g., pectinases, cellulases, hemicellulases), allowing the cell contents to be released. The use of lipases or proteases allows the degradation of cell membranes. Enzymatic treatment of microalgae cells ensures selective and efficient extraction of proteins while maintaining their integrity for various applications. The use of lipases or proteases allows the degradation of cell membranes. Enzymatic treatment of microalgal cells ensures selective and efficient extraction of proteins while maintaining their integrity for various applications. However, the high cost of the enzymes used can be a significant obstacle to their use on an industrial scale [126].

Following extraction, several centrifugation cycles are performed, and proteins are recovered through chromatographic, filtration, or precipitation techniques [125]. While some of these techniques yield satisfactory results, they also present drawbacks, such as the use of toxic solvents, high levels of contaminants, lengthy processing times, high costs, or specific conditions (e.g., high temperatures) that can denature the recovered proteins [127].

Proteins, being delicate molecules, require gentle extraction conditions to preserve their native structure and biological activity. Additionally, it is important to note that a one-step method is often insufficient, particularly for obtaining highly purified, high-concentration protein products. For example, Gifuni et al. [128] demonstrated that a triple-filtration process applied to *Chlorella sorokiniana* biomass achieved only 12% protein recovery. Although membrane chain filtration holds promise for the gentle handling of proteins, the technology remains in its early stages. It is likely that incorporating additional steps between cell wall disruption and filtration would significantly enhance protein recovery levels.

Currently, there is a trend toward multi-stage approaches [127,129]. Alternative or improved methods for protein recovery from microalgae cells include pulsed electric field (PEF)-assisted extraction, ultrasonic-assisted extraction (UAE), and microwave-assisted extraction (MAE) [123,130]. The use of acoustic energy in the UAE method allows for increased release rates and diffusion of target materials through solvent cavitation. UAE offers significant advantages, including minimal subsequent processing, ease of execution, low solvent consumption, and non-thermal characteristics, while enabling the recovery of large quantities of highly purified proteins [131]. For instance, Hildebrand et al. [132] reported a protein recovery rate of 79.1 ± 5.3% using UAE, which was 1.32 times higher compared to conditions without ultrasound. The addition of protease to the UAE process further enhanced protein recovery to 82%.

MAE, on the other hand, facilitates the efficient extraction of compounds, including proteins, while maintaining energy efficiency [130]. In a study by Motlagh et al. [133] on *Nannochloropsis* sp., MAE achieved a protein recovery rate of 26.35%, compared to just 0.63% with the Soxhlet method. This result underscores the superiority of microwave-assisted extraction, particularly when combined with ionic liquids, over traditional methods.

The PEF method is fast yet environmentally friendly, employing high electric currents to achieve perforation of the cell wall/cell membrane. Consequently, this leads to the release of microalgae cell contents. PEF enables the recovery of substantial amounts of proteins from *Chlorella vulgaris* while reducing processing costs by eliminating the need for energy-intensive biomass drying [123,134].

The cited examples demonstrate that protein extraction methods supported by ultrasound, microwave radiation, and pulsed electric fields are effective and efficient tools for recovering soluble proteins from microalgae biomass. Enhancing individual methods, such as combining UAE with enzymatic treatment or sequential solvent use, can increase yields but also incur additional costs. Therefore, selecting the most suitable method should be guided by specific needs and cost-effectiveness.

Many protein separation and concentration methods are challenging to scale due to high costs and time requirements (e.g., specialized equipment and skilled personnel). As a result, there is a demand for simple, fast, cost-effective, and easily scalable techniques. One such method is three-phase partitioning (TTP), which enables the recovery of large amounts of purified protein (90%) from crude extracts. In its standard form, TTP relies on protein precipitation using *t*-butanol and ammonium sulfate. Various adaptations of this technique have been developed, incorporating ultrasound, microwaves, ionic liquids, metal ions, enzymes, or macroaffinity ligands [127,135]. For instance, research on *Chlorella pyrenoidosa* demonstrated that optimizing TTP parameters, including enzyme pretreatment, significantly increased protein concentration [136]. Similarly, Chia et al. [127] found that combining TTP with sonication significantly improved protein yields (56.57–74.59%). Given its cost savings (70%) compared to standard chromatography, TTP is a viable option for industrial-scale applications [135].

Efforts to reduce protein extraction costs from microalgae cells also focus on optimizing solvent selection during the initial stages of the process to facilitate protein release from disrupted cells. Show et al. [137] evaluated various solvents, including methanol, ethanol, 1-propanol, and water, and found water to be the most effective. Recently, a single-stage method combining high shear mixing (HSM) with a liquid biphasic system (LBS) has been proposed to address challenges such as low efficiency and high costs. This method offers a quick, simple, and ecofriendly solution, utilizing low-toxicity salts [138]. According to the authors, this approach is effective, energy-efficient, and scalable for continuous processes due to its flexible configuration of individual parameters.

Although methods of protein extraction from microalgae have been researched and developed for many years, considering their advantages and disadvantages, the extraction of high-quality protein still remains a technological challenge.

## 6. Trials and Commercialization of Biopharmaceuticals

The unique characteristics of microalgae have made them suitable for numerous industrial applications, including feed, cosmetics, food, fertilizers, biofuels, wastewater treatment, and health products. However, the current market for modified microalgae primarily focuses on optimizing biomass production, with a strong emphasis on enhancing the biosynthesis of lipids, carbohydrates, and proteins for nutritious food. This trend is not surprising, as the commercial cultivation of microalgal biomass has been developing for 60 years—initiated by companies in Japan and Taiwan—and is now predominantly concentrated in the USA and Asia. In Europe, Germany appears to be the largest player in this sector [139,140]. According to FAOSTAT data from 2021, global algae production increased 60 times from 1950 to 2019 [141]. The dominance of biomass production is evident both in scientific publications (e.g., Camacho et al. [140], Hu et al. [142]) and in the activities of companies and startups. In December 2024, Intellectual Market Insights Research identified the top 10 leading companies in the Global Microalgae Market, including Corbion, DIC Corporation, Cyanotech Corporation, Cellana, AlgaEnergy, Algaecytes, Allmicroalgae, Parry Nutraceuticals, Euglena, and Kuehnle AgroSystems (https://www.intellectualmarketinsights.com, accessed on 5 January 2025) [143]. These companies specialize in producing high-value natural products for functional foods, feed, cosmetics, biofuels, and environmental solutions. Meanwhile, numerous studies have reported the successful synthesis of various types of heterologous recombinant therapeutic proteins derived from microalgae-based systems (examples listed in Table 1). However, no such biopharmaceuticals have yet reached the market [144,145]. This includes protein therapeutics dedicated to human therapy, exogenous to microalgae, which we focused on in our review. Among other factors, the lack of approval for commercial production was due to (1) limited market size; (2) production at non-competitive costs compared to alternative products obtained through chemical synthesis, metabolism by other microorganisms, or even extracts from fossil raw materials; and (3) stricter regulatory requirements regarding quality standards, safety assurance, and reduction in environmental impact [140]. The latter two constraints especially apply to the biologics market, which definitely is not limited but is demanding and dynamic. The cost-related challenges of algal protein production arise from the interplay of high manufacturing expenses, technological constraints, market conditions, and regulatory compliance demands. Although the FDA considers microalgae safe, the list of edible GRAS species remains limited. The situation in the EU is even more restrictive, with stricter regulatory requirements, particularly in the context of human therapeutic applications. Above all, certain challenges are associated with the availability of genetic tools, the limited number of sequenced genomes, and the highly specific genetic background of microalgae species (as discussed in Section 4). As a result, only a few projects have reached more advanced stages of development, including preclinical and clinical trial phases. An analysis of industrial start-ups revealed several projects focused on utilizing microalgae for biologics production for human therapy (StartUs Insights Discovery Platform) [146]. An especially interesting example is the aforementioned Lumen Bioscience, a start-up founded in 2017, which develops dual-purpose products serving both as a source of biologics and biomass. Lumen Bioscience Inc. employs genetically modified *A. platensis* (previously *Spirulina*) in the production of edible biologics, as spirulina cells are able to express far higher amounts of therapeutic proteins than any other food crop and are a promising vaccine delivery system. Their product pipelines include, among others, inflammatory bowel disease, kidney stone disease, and *Clostridium difficile* infections. Additionally, they are developing an intranasal/oral spirulina-based PfCSP malaria vaccine [61], currently at the preclinical stage, and antibody cocktails for gastroenteritis, presently undergoing pharmacokinetic studies (www.lumen.bio, accessed on 4 February 2025) [147]. According to various papers, approximately 50 recombinant proteins have been efficiently produced in microalgae [76]. Several examples of preclinical trials involving recombinant proteins expressed in microalgae have been reported [74]. However, the reported numbers reflect a broader definition of biopharmaceuticals, as various studies include not only exogenous proteins but also endogenous proteins and/or those developed for animal therapeutic applications. Additionally, tracking the development of microalgae-based drugs poses a challenge, as it may be too early to expect the presence of candidates from preclinical trials, such as those described in studies from 2016 [74], on the market. A particularly valuable approach would be to screen world clinical trial databases (e.g., ClinicalTrials.gov, covering studies conducted in the USA and over 200 countries) to evaluate the level of development of therapeutic exogenous recombinant proteins derived from microalgae tested in humans (https://clinicaltrials.gov, accessed on 4 February 2025) [148].

The successful commercialization of microalgae-based biopharmaceuticals will require a multidisciplinary approach combining advances in genetic engineering, bioprocess optimization, and regulatory science. Investment in genomic resources, high-throughput screening technologies, and bioreactor design will be crucial to overcoming current limitations. Furthermore, fostering collaboration between academia, industry, and regulatory bodies could facilitate the development of consistent quality standards and accelerate the transition of microalgae-derived biologics from bench to bedside.

## 7. Conclusions

Microalgae present a viable and promising platform for the production of human therapeutic recombinant proteins. Their advantages include cost-effectiveness, scalability, the ability to perform necessary post-translational modifications, and immunity to human pathogens. These features, coupled with the development of well-established transformation methods and optimized vectors, have enabled the successful expression of various biopharmaceuticals, ranging from vaccines to enzymes. Additionally, the potential to engineer edible biopharmaceuticals in microalgae offers an innovative approach to oral drug delivery, eliminating the need for complex purification steps and reducing production costs. However, despite significant advancements, the efficacy of protein production remains a major challenge. Low transgene expression, limited protein accumulation, and constraints related to post-translational modifications in plastidic expression must be further addressed to fully unlock the potential of microalgae as biofactories.

A major emphasis of this work is on the production of heterologous recombinant proteins specifically designed for human therapy. Compared to conventional platforms, microalgae offer a sustainable alternative that can be tailored for large-scale pharmaceutical applications. Nevertheless, future research should focus on integrating innovative technologies to improve process scalability, reduce costs, and enhance the sustainability of cultivation methods. In the case of genetic engineering, further screening for more efficient regulatory sequences, the expansion of the number of investigated and transformed algal species, and improvements in synthetic biology approaches—such as optimized promoters, engineered vectors, and advanced genome-editing techniques—are required.

As the demand for protein-based therapeutics continues to grow, microalgae have the potential to emerge as a critical component of the global biopharmaceutical landscape. Moreover, ensuring that microalgal-based pharmaceuticals meet GRAS requirements is crucial for their regulatory approval and commercial application. Despite their immense promise, the transition from laboratory-scale research to full-scale commercial production remains a major hurdle. Addressing regulatory challenges, optimizing cost-effectiveness, and securing industrial partnerships will be key to unlocking the commercial viability of microalgal biopharmaceuticals. Collaboration between academia, industry, and regulatory agencies will be essential to streamline approval processes and accelerate market entry. By overcoming these obstacles, the field can advance towards more efficient and economical production of biopharmaceuticals, ultimately contributing to more accessible and cost-effective therapies for a wide range of diseases.

## Figures and Tables

**Table 1:** Achievements in recombinant protein production in microalgae over the last ten years.

Species	Localization	Product	Treatment	Tests	Results	Refs.
*P. tricornutum*	nucleus	monoclonal IgG antibodies against the nucleoprotein of the Marburg virus	Antivirus	Western blot, ELISA test	Microalgae-produced antibodies with functionality comparable to hybridoma-produced antibodies	[50]
*Chlamydomonas reinhardtii*	nucleus	Human interferon-α	Immunity	In vitro and in vivo antitumor and antiviral assay properties Cytotoxicity and cell apoptosis assays)	Suppression of tumor growth (in Hep-G2 tumor cell lines); antiviral activity against vesicular stomatitis virus (VSV).	[51]
*C. reinhardtii*	nucleus	Human lactoferrin (hLF)	Antimicrobial	In vitro and in vivo tests	Significant antibacterial activity and little toxicity to mice	[52]
*Chlorella* sp.	nucleus	Human lactoferrin (hLF)	Antimicrobial	inverted fluorescence microscope	accumulation of human lactoferrin	[53]
*Schizochytrium* sp.	nucleus	Brest cancer tumor epitopes (BCB)	Breast cancer	In vivo tests on mice/serological test	Immunogenic activity algae-made BCB	[54]
*C. reinhardtii*	nucleus	Vascular endothelial growth factor (VEGF 165)	Pro-angiogenic growth factors in wound healing approaches	In vitro angiogenesis assays	Efficient production of recombinant protein	[55]
*C. vulgaris*	nucleus	Human granulocyte-colony stimulating factor (hG-CSF)	Stimulation of bone marrow for increased cell production used during as well as after chemotherapy	Western blot detection	Efficient production of recombinant protein	[56]
*Haematococcus pluvialis*	chloroplast	Antimicrobial peptide (AMP)—piscidin-4	Antimicrobial	Western blot detection	Accumulation of recombinant antimicrobial peptide piscidin-4	[57]
*C. reinhardtii*	chloroplast and nucleus	Receptor-binding domain (RBD) of the SARS-CoV-2 spike protein	Antiviruses	Western blot detection; ACE2 receptor binding interaction assays; serological tests	Correctly folded and functional SARS-CoV-2 spike protein RBD	[58]
*C. reinhardtii* *Chlorella vulgaris*	nucleus	SARS-CoV-2 receptor binding domain (RBD); basic fibroblast growth factor (bFGF)	Antiviruses, tissue repair	Western blot detection; Elisa test	Efficient production of recombinant proteins	[59]
*Phaeodactylum tricornutum*	nucleus	Receptor-binding domain (RBD) of the SARS-CoV-2 spike protein	Antiviruses	Western blot detection and in vitro tests	Production of SARS-CoV-2 or other coronavirus antigens for pandemic diagnostics	[60]
*A. platensis*	nucleus	PfCSP vaccine (against malaria)	antiparasitic	ELISA test	strong, systemic anti-PfCSP immune response	[61]
*C. reinhardtii*	chloroplast	Interleukin 29 (IL29)	Antitumor	Anti-proliferating bioassay using HepG2 cells.	Inhibition of HepG2 cell growth by IL29	[42]
*C. reinhardtii*	nucleus	Human protein α-Klotho (α-KL)	Treatment of various diseases (cancers, chronic kidney disease, atherosclerosis, etc.)	Antitumor activitis using *Rattus norvegicus* AR42J pancreatic tumor cell lines	Anticarcinogenic activity of recombinant mα-KL was confirmed	[62]
*Schizochytrium* sp.	nucleus	Tc24-CO1	Chagas disease	In vivo tests (mice immunized orally)	Immunogenic activity	[63]
*C. reinhardtii*		Multifunctional peptide BmKbpp	Antimicrobial	In vitro antibacterial tests	Inhibitory effects on the growth of Gram-positive bacteria	[64]
*Porphyridium purpureum*	nucleus	HCV E2 glycoprotein	A candidate vaccine against the hepatitis C virus (HCV)	In vivo tests (mice immunized injection and orally)	Immunogenicity of the HCV antigen	[65]
